# Reliability and validity of the Arabic version of the Early Onset Scoliosis 24 Items Questionnaire (EOSQ-24)

**DOI:** 10.1051/sicotj/2019001

**Published:** 2019-02-08

**Authors:** Yahia Hanbali, Tony Perry, Asif Hanif, Hiroko Matsomotu, Haytham Musmar, Khaldoun Bader, Alaaeldin Azmi Ahmad

**Affiliations:** 1 Faculty of Medicine and Health Sciences, An-Najah National University Nablus 41414 Palestine; 2 Assistant Program Manager, Waltham Forest Clinical Commissioning Group London E11 UK; 3 Asst. Prof. & Head of Department, Bio-Statistics, GD-PGMI 54000 Pakistan; 4 Department of Orthopaedic Surgery, Columbia University Medical Center 630 West 168th Street, #1504 10032 New York City NY USA; 5 Assistant Professor in Epidemiology, School of Public Health, Al-Quds University 90612 Palestine; 6 Pediatric Orthopedic Surgeon, Associate Professor, Poly Technique University-Palestine PO Box 3985 Ramallah, West bank 602 Palestine

**Keywords:** Early onset scoliosis, Arabic version, Early Onset Scoliosis-24 Questionnaire, Health-related quality of life, Validation, Validation

## Abstract

*Introduction*: Early Onset Scoliosis (EOS) is a complex pathology that covers a variety of etiologies, with onset before the age of 10 years. Surgical treatment of EOS should have the objectives of fulfilling maximum pulmonary function, spine length, with minimal hospitalizations, complications, and family burden. Radiographic parameters are an important standard in assessing treatment outcomes. However, the Early Onset Scoliosis Questionnaire-24 (EOSQ-24) was developed to measure the wider dimensions of outcomes involving the quality of life of patients and caregivers post-treatment. The aim of this study was to evaluate the validity and reliability of culturally adapted Arabic version of the EOSQ-24.

*Methods:* Translation and cross-cultural adaptation, based on published guidelines, were performed on the original English EOSQ-24 by a committee. The Arabic version of EOSQ-24 was applied to the caregivers of all 58 EOS patients who were treated surgically after signing a consent form. Reliability was assessed using Cronbach’s *α* and item-total statistics for the whole questionnaire initially and for the each domain separately. Data quality was assessed by mean, median, percentage of missing data, ceiling and floor effects. Discriminative validity was examined using non parametric tests.

*Results*: The response for all items was excellent with only 1.7% (0–1) of responses missing. The floor effect ranged from 0% to 36.2% of patients and the ceiling effect ranged from 0 to 46.6%. Cronbach’s *α* test reliability was found excellent (0.919), as was the internal consistency of all domains, with Cronbach *α* ranging from 0.903 to 0.918. Corrected item-total correlations were good for all domains (>0.3). Only one item (Question 21) showed low corrected item-total correlations (*r* = 0.222). However, Cronbach’s *α* did not increase significantly when this item was deleted (0.920).

*Conclusion*: The first adapted Arabic version of EOSQ-24 is found to have good validity and reliability, and it can be used to assess children in Arab societies with EOS.

## Introduction

Early Onset Scoliosis (EOS) is a complex pathology that covers a variety of etiologies including idiopathic, neuromuscular, syndromic, and congenital types, with onset before the age of 10 years [1]. Its natural history is associated with progressive deterioration in pulmonary function and poor quality of life. In addition, EOS patients may have associated pathological cardiopulmonary and gastrointestinal conditions that exacerbate the burden of spinal deformity on pulmonary function, if left untreated [2,3].

Current options for the treatment of EOS aim to stop the progression of curve as well as give the spine and the thorax an opportunity to grow [4]. Technological advances have significantly improved the growth-friendly spinal implants, which aim to correct the abnormal curvature, while maintaining the growth of the spine and the thorax. The safety and efficacy of growth-friendly techniques in the treatment of EOS, in addition to improvements in the quality of life in EOS patients, have been documented in the literature [5,6]. Growing instrumentation “growing rod” (GR) and “vertical expandable prosthetic titanium rib” (VEPTR) are spine-based and rib-based distractions, respectively, utilized as an alternative to arthrodesis [7,8]. These growing methods have shown promising results, although they have negative impacts on the health of children due to repetitive surgeries [9–11].

For many years, the assessment of quality of life for EOS patients depended on radiographic parameters that show progression of curve. However, with a growing interest on the impact of treatment on health-related quality of life, outcome measures that assess the subjective response of patients to their disease, as well as the burden on the caregivers, are considered an important part of health assessment [12,13].

Matsumoto et al. [14] developed Early Onset Scoliosis-24 Questionnaire (EOSQ-24) as a tool for evaluation of both patients and parents. EOSQ-24 showed a picture of quality of life pre and post operatively. The questionnaire was developed in English language, and after it showed good internal consistency and reliability, it was modified into its current version [15].

EOSQ-24 needs to be culturally adapted to retain consistency and validity for the assessment of a new population. This is particularly important for uncommon disorders, such as EOS, where multicenter multinational cooperation is fundamental to amass a larger patient series and compare results at a multicenter level [16].

Previous studies have shown the validity and reliability of EOSQ-24, either in its original language [14] or after it was translated into Turkish, Spanish, and Chinese languages [17–19]. The authors recognized a need to create an Arabic version of the EOSQ-24 [14].

The aim of this study was to translate the original EOSQ-24 into Arabic, and to test the reliability and discriminative validity of the Arabic version of EOSQ-24. Discriminative validity was used since there is no valid questionnaire in Arabic that deals with children’s health.

## Methods

The Early Onset Scoliosis-24 (EOSQ-24) is a survey instrument used to assess the quality of life of children who have early onset scoliosis (EOS). It is a subjective questionnaire completed by parents on behalf of child patients. The EOSQ-24 consists of 24 questions with 11 domains: general health, pain, pulmonary function, transfer, physical function, daily living, fatigue, emotion, parental burden, financial burden, and satisfaction [14].

We ran through all the files for the cases with EOS that had surgery done for management.

All of the families were contacted and the questionnaire was filled either personally or by phone. It took one year to complete the data (2016).

### Study design

The authors contacted the original developers to confirm that no other groups were translating the EOSQ-24 into the Arabic language, and obtained permission to carry out this translation.

There were two different stages for this study: First, the EOSQ-24 was translated into Arabic and transculturally adapted, following the best practice guidelines described by Beaton et al. [20] and accepted by the developers of the questionnaire. Second, the information was collected from patients’ caregivers. All ethical considerations were taken care of by the ethics committee of An-Najah National University, and a consent form was signed by parents of each child.

#### Cross-cultural adaptation and translation process

The questionnaire was translated from English to Arabic by two native Arabic speakers fluent in English, one an orthopedist and the other with no medical background. A third independent committee reviewed both translations. The committee was comprised of a pediatric orthopedic surgeon, two orthopedic surgeons, and translators to find discrepancies between the two translation to create a pretesting Arabic version of the EOSQ-24.

The pretesting Arabic form was tested on a sample of patients to prove that they could understand each question clearly and to make changes to questions that were not clear. The form was then translated back by two native English speakers with good Arabic knowledge. Both were blinded to the purpose of the study and had no access to the original questionnaire. The committee finalized the pretesting form after discussing all translations, and added modifications to any ambiguous expressions.

The final version was tested on the entire available sample by interviewing the 58 parents of children. We asked them if the questions were relevant to their child’s condition and confirmed their understanding of the questions.

### Data analysis

Each question in the Arabic version of questionnaire was scored on a 5-point Likert scale, with higher scores indicating lower disability. Moreover, a score for each domain was calculated. All statistical analyses were performed using SPSS (Statistical Package for the Social Sciences) software version 20.0. Categorical variables were represented by percentages, while continuous variables were reported as median and interquartile ranges. Internal consistency, validity, and ceiling and floor effects were also analyzed in this study.

#### Internal consistency

Internal consistency was assessed using the Cronbach *α* coefficient, which is used to measure how closely related questions within a survey or a given subset of survey questions are. This is consistent with the approach taken for testing the original EOSQ-24 survey. The recommended value for Cronbach *α* is between 0.70 and 0.95 [21].

Internal consistency was also assessed using Item-total correlations and inter-item correlations. Item-total correlations explore how each item is related to other items in the scale; 0.3 or higher is considered good correlation in the domain [21–23]. Inter-item correlations give an indication that the items are too similar; a good range for inter-item correlations is between 0.2 and 0.8 [21–23].

#### Ceiling and floor effects

Ceiling and floor effects were used to determine the ability of the questionnaire to assess severity condition, while descriptive statistics (mean values, and quartiles) were calculated to show distribution for domains. Analysis was done by calculating the frequency of extreme possible scores, with values less than 30% considered acceptable [24].

#### Discriminant validity

Discriminant validity was tested for each domain with a range of combinations of patient data, including: Radiographical indices (Cobb angle before surgery), gender, type of Scoliosis (congenital, idiopathic, neuromuscular, and syndromic), ambulatory status, and whether there were complications. The non-parametric Kruskal–Wallis and Mann–Whitney *U* tests were used to compare between different groups of patients, while the Spearman correlation coefficient was used to find correlation between quantitative parameters. *P* values less than 0.05 were considered statically significant.

## Results

The 58 caregivers completed the EOSQ-24 questionnaire after they agreed to participate, and signed a consent form explaining the objective of the survey. The caregivers completed the surveys. The clinical and demographic characteristics are summarized in Table 1.

**Table 1:** Characteristics of patients surveyed using EOSQ-24.

Characteristic	Value (number of patients)
Gender	
Male %	32.76% (19)
Female %	67.24% (39)
Median age at surgery	62 months
Complications %	
Yes	50
No	50
Diagnosis %	
Congenital	39.66% (23)
Syndromic	25.86% (15)
Neuromuscular	22.41% (13)
Idiopathic	12.07% (7)
Ambulatory %	
Yes	91.4% (53)
No	8.6% (5)
Site of main curve %	
Thoracic	36.2% (21)
Thoracolumbar	58.6% (34)
Lumbar	5.2% (3)
Number of Curves %	
1	44.8% (26)
2	48.3% (28)
3	6.9% (4)

### Translation and transcultural adaptation

There were some cases where translators had difficulty identifying a conceptually equivalent word to capture the meaning of the original question. Questions 8 and 12 were particularly difficult, so the wording of these questions was reviewed and tested carefully to ensure their meaning was clear and conceptually consistent with the original English version.

#### Internal consistency

Cronbach’s *α* was used to find the internal consistency of all items of questionnaire and of each domain separately, which included: general health, pain, pulmonary function, mobility, physical function, daily living, fatigue, emotion, parental burden, financial burden, and satisfaction.

Cronbach’s *α* for all items showed an excellent overall reliability with 0.919. Corrected item-total correlation was also calculated for each question, and the results were within the acceptable range (0.357–0.735), except for question 21 (0.222). Cronbach’s *α* if Item Deleted ranged between 0.912 and 0.920 and Cronbach’s *α* if Item Deleted for the domain ranged between 0.903 and 0.918 (Table 2).

**Table 2:** Tests for Internal Consistency.

Domains	Scale mean if item deleted	Scale variance if item deleted	Corrected item-total correlation	Cronbach’s *α* if item deleted
General health				.912
Q1	79.53	237.735	.483	.917
Q2	79.24	237.628	.655	.914
Pain/discomfort				.913
Q3	79.42	234.063	.601	.914
Q4	79.20	237.459	.555	.915
Pulmonary function				.905
Q5	78.64	233.236	.721	.912
Q6	79.11	232.247	.713	.912
Transfer				.913
Q7	79.07	229.032	.665	.913
Physical function				.903
Q8	79.31	238.662	.525	.916
Q9	78.82	231.114	.677	.913
Q10	79.18	231.929	.588	.915
Daily living				.912
Q11	79.33	234.928	.509	.916
Q12	79.35	232.786	.578	.915
Fatigue/energy level				.906
Q13	79.47	235.291	.735	.913
Q14	79.18	234.226	.680	.913
Emotion				.918
Q15	79.67	242.224	.357	.919
Q16	78.98	239.685	.459	.917
Parental impact				.913
Q17	80.76	239.295	.544	.915
Q18	79.00	241.926	.445	.917
Q19	79.16	244.251	.435	.917
Q20	79.11	245.136	.458	.917
Q21	79.25	251.082	.222	.920
Financial impact				.917
Q22	80.20	238.607	.445	.917
Satisfaction				.914
Q23	79.80	240.607	.464	.917
Q24	79.62	231.759	.562	.915
			α	0.919*

* Alpha coefficients for the total EOSQ scales.

#### Ceiling and floor effects

Ceiling and Floor effects were calculated to ensure that the questions had sufficient variability for differentiating between responses and adequately measuring correlations between variables. Ceiling and floor effects below 30% were considered acceptable. Several of the questions (notably questions 5, 6, 7, 9, 10, and 18) exceeded the threshold for ceiling effects, but all questions met the threshold for floor effects. This resulted in a floor effect in 0%–36.2% of patients and a ceiling effect in 0%–46.6% (Table 3), and may reflect a higher severity of disease for the children tested at this hospital in Palestine.

**Table 3:** Ceiling and floor effects of Arabic EOSQ-24 (*n* = 58).

Domain	Response	Missing %	Mean	Median	Ceiling	Floor
General health			61	62.5		
Q1	58	0	3.3	3.0	17.2%	8.6%
Q2	58	0	3.6	4.0	10.3%	5.2%
Pain/discomfort			64.2	62.5		
Q3	58	0	3.5	3.5	24.1%	8.6%
Q4	57	1.7	3.6	4.0	29.8%	3.5%
Pulmonary function			72.8	75		
Q5	58	0	4.1	4.0	46.6%	3.4%
Q6	58	0	3.7	4.0	32.8%	1.7%
Transfer			68.1	75		
Q7	58	0	3.7	4.0	34.5%	10.3%
Physical function			68.8	75		
Q8	58	0	3.6	4.0	20.7%	5.2%
Q9	58	0	4.0	4.0	43.1%	6.9%
Q10	57	1.7	3.6	4.0	33.3%	8.8%
Daily living			62.1	68.8		
Q11	58	0	3.5	4.0	27.6%	12.1%
Q12	58	0	3.5	4.0	24.1%	10.3%
Fatigue/energy level			61.6	62.5		
Q13	58	0	3.3	3.0	10.3%	3.4%
Q14	58	0	3.6	4.0	25.9%	1.7%
Emotion			62.3	62.5		
Q15	57	1.7	3.1	3.0	14.0%	12.3%
Q16	57	1.7	3.8	4.0	38.6%	3.5%
Parental impact			57.9	60		
Q17	58	0	2.0	2.0	3.4%	36.2%
Q18	58	0	3.8	4.0	31.0%	1.7%
Q19	58	0	3.6	4.0	13.8%	5.2%
Q20	58	0	3.7	4.0	13.8%	0.0%
Q21	58	0	3.6	4.0	0.0%	5.2%
Financial impact			40.5	25		
Q22	58	0	2.6	2.0	8.6%	20.7%
Satisfaction			53.9	50		
Q23	57	1.7	3.0	3.0	10.5%	5.3%
Q24	58	0	3.3	4.0	24.1%	12.1%

#### Response distribution

Median scores for questions ranged between 2.0 (Q17) and 4.1 (Q5), while the mean score of domains were between 40.5 (Financial Impact) and 72.8 (Pulmonary Function), as shown in Table 3. This is based on a very high responsiveness to questions; only 1.7% of questions had missing answers.

#### Discriminative validity

**Figure 1:**
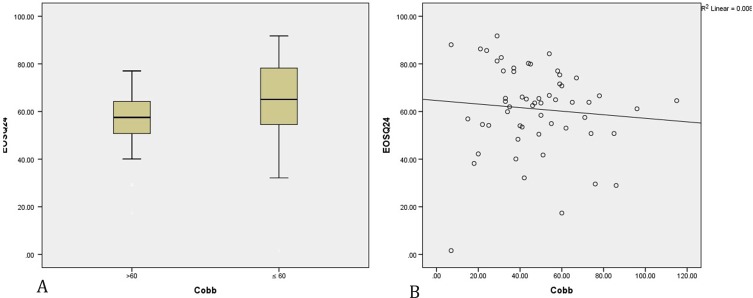
(A) Comparison of total scores between EOS patients with Cobb angle ≤60° and >60° were performed using Mann–Whitney *U* test. (B) Correlation between total scores and Cobb angles were evaluated using a Spearman rank correlation coefficient test.

In this study, we analyzed variables that had an association with the scores of the Arabic version of EOSQ-24. Patients involved in this study had severe curves, so 60° was set as the cutoff-point for radiographical indices (Cobb scores). Patients with curves below 60° had much better scores in comparison to patients with curves above 60° (*p* = 0.039); Figure 1A. Moreover, we examined Spearman’s correlation and found a weak negative correlation between the total score of Arabic version of EOSQ-24 and Cobb angles. (*ρ* = −0.276 with *p* = 0.038); Figure 1B.

Regarding complications, we found that patients who had complications after surgery had lower scores when compared to patients who had not (*p* = 0.035). Finally, regarding ambulatory status of patients, we found higher scores in ambulatory patients compared to non-ambulatory patients (*p* = 0.02). No significant results were found for other variables.

## Discussion

Although EOS is considered an uncommon illness, early treatment is mandatory. If left untreated, EOS will lead to increased severity of curves, which increases pulmonary morbidity and cardiovascular problems due to lack of development of the trunk. Quality of life as well as life expectancy decreases dramatically for untreated patients. Surgical treatment options aim to halt progression of curves and maintain the ability of the spine to continue growing, with positive effects on respiratory systems [4, 9, 25]. On the other hand, treatment options may have negative effects on the quality of health if they involve repetitive surgeries [5,11]. Matsumoto et al. also found negative effects on the psychosocial aspects for the patient and the family [26]. Therefore, it was necessary to find instruments to assess health-related quality of life of patients and their caregivers.

For many years, in part because of heterogeneity of this population, there was no specific tool to assess the Health-Related Quality of Life (HRQoL) for EOS patients and the burden on their caregiver. The EOSQ-24 survey was designed to meet this need [9,10], and was originally developed in English.

The Arabic language is an official language in 22 countries with more than 400 million people. To the best of our knowledge, this is the first study that describes cross-cultural adaption and translation of the EOSQ-24 survey into the Arabic language. The survey was translated into Arabic using a rigorous process of translation, back-translation, and testing for reliability and discriminative validity. Our results show excellent reliability as well as an assessment of cross-sectional differences according to several clinical features, such as Cobb angles, ambulatory status, and complications.

Cronbach’s *α* was used to test the internal consistency of the Arabic Version of EOSQ-24, which was very good across the 24 items and 11 domains. The internal consistency for the original EOSQ-24, as measured by Cronbach’s *α*, was 0.92 [14]. By comparison, the internal consistency of our Arabic version, as measured by Cronbach’s *α*, was 0.919. This is much closer to the original version than other translated versions of questionnaire, which include Chinese (Cronbach *α*: 0.896), Turkish (Cronbach *α*: 0.909), and Spanish (Cronbach *α*: 0.897) versions.

Cronbach *α* of items deleted for each question in our study varied from 0.912 to 0.920, while in Turkish it ranged from 0.902 to 0.908 and in Spanish from 0.802 to 0.898. Moreover, the corrected item-total correlation was 0.357–0.735 in our study with only one question below 0.3. By contrast, in Spanish the correlation ranged between 0.354 and 0.675 with two questions below 0.3, while in Turkish it ranged between 0.272 and 0.698 with one question below 0.3.

With respect to floor and ceiling effects, our study revealed a floor effect in 0–36.2% of patients and a ceiling effect in 0–46.6%. This compares favorably to the other language versions of EOSQ-24. The floor effect in the Turkish version ranged from 0 to 21.7% of patients, while the ceiling effect ranged from 1.6% to 68.3%. In the Spanish version, the floor ranged from 0 to 29.5% and the ceiling ranged from 9.1% to 74.4%. While the Chinese version had a floor range of 0–26%, and a ceiling range of 0–71%.

The correlation between total scores and Cobb angles in our study was *ρ* = −0.276 with *p* = 0.038; this is considered weak but is still an acceptable correlation. In the Spanish study, *ρ* = −0.473 with *p* = 0.004, was considered a moderate correlation.

The item-total correlations were found able to show discriminative validity for all questions except Q21, which is regarding parental burden. Q21 also had the lowest reliability (0.222) mostly due to heterogeneity of population and a more severe form of scoliosis. Both Q5 and Q21 had low reliability; that was also due to the heterogenicity for Q21, and the severity of the scoliosis in all cases

Similar to the Spanish study, the Arabic version of EOSQ-24 was able to discriminate between EOS patients according to severity of curves, complications after surgery, and ambulatory status. As all participants in the study were treated surgically because of severe curvature, we found that the increment in curve will lower the total score, through a negative relation between score and Cobb angles. In addition, patients who had complications after surgery had a lower score. Non-ambulatory patients also had lower total scores, though this was questionable because only 8.6% of participants were non-ambulatory.

The heterogeneity of our EOS population, consisting of patients of various ages, disorders, and stages of treatment, can explain the wide range of distribution of scores among parents’ answers. Ceiling effects were found in some questions, which concern pulmonary function and physical aspects. This may be due to the severity of curvature that required surgical treatment in all cases.

### Limitations of the study

1. It is an experience of one center with one surgeon’s work.
2. All patients were surgical cases with severe scoliosis.
3. All patients had the same insurance and most of them had similar socioeconomic status.

## Conclusion

The Arabic version of EOSQ-24 provides a reliable tool to assess children with EOS. However, acknowledging the limitations of this study, future studies should be conducted with multicenter data with fewer variables and more homogenous diagnosis.

## Conflict of interest

All authors declare that they have no conflict of interest.

## Supplemental material

Valid and Adapted Arabic translation of the original English Early Onset Scoliosis Questionnaire EOSQ24. Click here for additional data file. The Supplemental material is available at https://sicot-j.org/10.1051/sicotj/2019001/olm.
