# Supplementary material for: Reliability and validity of the Arabic version of the Early Onset Scoliosis 24 Items Questionnaire (EOSQ-24)
Source: SICOT J. 2019 Feb 8;5:7. doi: 10.1051/sicotj/2019001 (PMC6405253; doi:10.1051/sicotj/2019001)
Supplement: Supplementary file 1 — Valid and Adapted Arabic translation of the original English Early Onset Scoliosis Questionnaire EOSQ24. [file sicotj-5-7-s1.docx]

**انحناء العمود الفقري البادئ مبكراً**

**استبيان مكون من 24 سؤال**

**(EOSQ-24)**

**مركز ابحاث جراحة عظم الأطفال**

**مركز جامعة كولمبيا الطبي**

| الحالة الصحية العامة: خلال الأسابيع 4 السابقة | | | | |
| --- | --- | --- | --- | --- |
| 1. بشكل عام، كيف تقيم الحالة الصحية لطفلك؟ | | | | |
| ممتازة | جيدة جدا | جيدة | مقبولة | ضعيفة |
| 1. كم بالعادة يكون طفلك مريضاً؟ | | | | |
| دائماً | معظم الأوقات | بعض الاوقات | نادراً | أبدا |

| ألم/ عدم الراحة: خلال الأسابيع 4 السابقة | | | | |
| --- | --- | --- | --- | --- |
| 1. إلى اي مدى كان\كانت طفلك\طفلتك يعاني من ألم أو عدم الراحة؟ | | | | |
| دائماً | معظم الأوقات | بعض الاوقات | نادراً | أبدا |
| 1. كيف تقيم شدة هذه الألام؟ | | | | |
| شديد جداً | شديد | متوسط | ضعيف | لا ألم |

| الكفاءة الجاهز التنفسي: خلال الأسابيع 4 السابقة | | | | |
| --- | --- | --- | --- | --- |
| 1. ما مقدار الصعوبة التي واجهها طفلك\طفلتك في الكلام أو البكاء بدون حدوث ضيق في التنفس ( حسب عمر الطفل) ؟ | | | | |
| صعب | صعب نوعاً ما | محايد | سهل نوعاً ما | سهل |
| 1. كم بالعادة يعاني طفلك\طفلتك من ضيق في التنفس اثناء ممارسة نشاط معين؟ | | | | |
| دائماً | معظم الأوقات | بعض الاوقات | نادراً | أبدا |

| التنقلات: خلال الأسابيع 4 السابقة | | | | |
| --- | --- | --- | --- | --- |
| 1. إلى اي مدى كانت الحالة الصحية لطفلك\طفلتك تحول دون وصله/ها الى احد الأماكن؟ | | | | |
| دائماً | معظم الأوقات | بعض الاوقات | نادراً | أبدا |

**انظر الصفحة التالية من فضلك**

| الأداء البدني: خلال الأسابيع الأربعة السابقة | | | | | | | | | |
| --- | --- | --- | --- | --- | --- | --- | --- | --- | --- |
| 1. ما مدى الصعوبة التي واجهها طفلك\طفلتك في تحريك الجزء العلوي من جسمه\جسمها؟ | | | | | | | | | |
| صعب | | صعب نوعاً ما | | محايد | | سهل نوعاً ما | | سهل | |
| 1. ما مدى الصعوبة التي واجهها طفلك\طفلتك في اتخاذ وضعية الجلوس بدون مساعدة؟ | | | | | | | | | |
| صعب | | صعب نوعاً ما | | محايد | | سهل نوعاً ما | | سهل | |
| 1. ما مدى الصعوبة التي واجهها طفلك\طفلتك في الحفاظ على توازنه\توازنها اثناء الحبو، أو المشي ، او الركض ؟ | | | | | | | | | |
| صعب | | صعب نوعاً ما | | محايد | | سهل نوعاً ما | | سهل | |

| الحياة اليومية: خلال الأسابيع الأربعة السابقة | | | | | | | | | |
| --- | --- | --- | --- | --- | --- | --- | --- | --- | --- |
| 1. ما مدى الصعوبة التي واجهها طفلك\طفلتك في ارتداء الملابس أو مساعدته في ذلك؟   (أمثلة: المساعدة في خلع او ارتداء الملابس، ادخال الذراع قي كمة القميص أو الرجل في البنطلون أو المساعدة في استخدام السحاب، أو المشبك أو الأزرار) | | | | | | | | | |
| صعب | | صعب نوعاً ما | | محايد | | سهل نوعاً ما | | سهل | |
| 1. إن أكل كمية من الطعام يستلزم من طفلي قضاء وقت أطول من طفل سليم لأكل الكمية نفسها من الطعام. | | | | | | | | | |
| موافق بشدة | | موافق | | محايد | | غير موافق | | غير موافق بشدة | |

| مستوى الحيوية\الإرهاق: خلال الأسابيع الأربعة السابقة | | | | | | | | | |
| --- | --- | --- | --- | --- | --- | --- | --- | --- | --- |
| 1. إلى أي مدى شعر طفلك\طفلتك بالإرهاق؟ | | | | | | | | | |
| دائماً | | معظم الأوقات | | بعض الاوقات | | نادراً | | أبدا | |
| 1. ما مدى الصعوبة التي يواجهها طفلك\طفلتك في الحفاظ على نشاطه\نشاطها طوال اليوم؟ | | | | | | | | | |
| صعب | | صعب نوعاً ما | | محايد | | سهل نوعاً ما | | سهل | |

**انظر الصفحة التالية من فضلك**

| العاطفة: خلال الأسابيع الأربعة السابقة | | | | | | | | | |
| --- | --- | --- | --- | --- | --- | --- | --- | --- | --- |
| 1. إلى أي مدى شعر طفلك\طفلتك بالتوتر أو القلق تجاه حالته\حالتها الصحية؟ | | | | | | | | | |
| دائماً | | معظم الأوقات | | بعض الاوقات | | نادراً | | أبدا | |
| 1. إلى أي مدى شعر طفلك\طفلتك بالإحباط تجاه حالته\حالتها الصحية؟ | | | | | | | | | |
| دائماً | | معظم الأوقات | | بعض الاوقات | | نادراً | | أبدا | |

| الأثر على الأهل: خلال الأسابيع الأربعة السابقة | | | | | | | | | |
| --- | --- | --- | --- | --- | --- | --- | --- | --- | --- |
| 1. إلى أي مدى شعرت بالقلق أو التوتر تجاه حالة طفلك\طفلتك الصحية؟ | | | | | | | | | |
| دائماً | | معظم الأوقات | | بعض الاوقات | | نادراً | | أبدا | |
| 1. إلى أي مدى تعراضت حالة طفلك\طفلتك مع أنشطة العائلة؟ | | | | | | | | | |
| دائماً | | معظم الأوقات | | بعض الاوقات | | نادراً | | أبدا | |
| 1. ما مدى تأثير حالة طفلك\طفلتك على مستوى حيويتك؟ | | | | | | | | | |
| شديد للغاية | | شديد | | ملحوظ بعض الشيء | | قليل | | غير ملحوظ | |
| 1. ما مدى تكرار تفويتك أو تأخرك عن العمل أو نشاط اجتماعي اخر نتيجة لحالة طفلك\طفلتك الصحية؟ | | | | | | | | | |
| دائماً | | معظم الأوقات | | بعض الاوقات | | نادراً | | أبدا | |
| 1. هل كنت قادرا\قادرة على قضاء وقت كاف مع العائلة\الزوجة على الرغم من حالة طفلك\طفلتك الصحية؟ | | | | | | | | | |
| دائماً | | معظم الأوقات | | بعض الاوقات | | نادراً | | أبدا | |

| التأثير المالي: خلال الأسابيع الأربعة السابقة | | | | |
| --- | --- | --- | --- | --- |
| 1. ما مدى العبء المالي الذي نتج بعد تشخيص طفلك\طفلتك بالانحناء الجانبي البادئ مبكرا ؟ | | | | |
| شديد | الى حد كبير | متوسط | قليل | لا يوجد عبء |

**انظر الصفحة التالية من فضلك**

| درجة الرضا: خلال الأسابيع الأربعة السابقة | | | | | | | | | |
| --- | --- | --- | --- | --- | --- | --- | --- | --- | --- |
| 1. إلى أي مدى شعر طفلك\طفلتك بالرضا تجاه قدرته/ قدرتها على القيام بالأعمال؟ | | | | | | | | | |
| غير راضٍ بتاتاً | | غير راضٍ | | محايد | | راضٍ | | راضٍ بشكل تام | |
| 1. إلى أي مدى تشعر انت بالرضا تجاه قدرة طفلك على القيام بالأعمال؟ | | | | | | | | | |
| غير راضٍ بتاتاً | | غير راضٍ | | محايد | | راضٍ | | راضٍ بشكل تام | |
